# Lifestyles and Pre-eclampsia with Special Attention to Cigarette Smoking

**DOI:** 10.2188/jea.13.90

**Published:** 2007-12-30

**Authors:** Akiko Ioka, Hideaki Tsukuma, Karo Nakamuro

**Affiliations:** 1 Department of Obstetrics and Gynecology, Osaka Prefectural General Hospital.; 2 Department of Cancer Control and Statistics, Osaka Medical Center for Cancer and Cardiovascular Diseases.

**Keywords:** lifestyles, cigarette smoking, household smoking exposure, alcohol drinking, pre-eclampsia

## Abstract

Cigarette smoking has been reported to protect women against pre-eclampsia. We conducted a cohort study of 493 women whose first antenatal visits were between September 1997 and April 1998 at Osaka Prefectural General Hospital, Japan. A self-administered questionnaire survey for lifestyles was carried out during pregnancy, and pregnancy outcome information was taken from medical record data. Of 493 subjects, 82 cases (16.6%) developed mild pre-eclampsia and 3 cases (0.6%) developed severe one. The prevalence of cigarette smokers decreased from 21.3% to 8.6% during early pregnancy. The incidence rate of pre-eclampsia among smokers was slightly greater than that among non-smokers (19.4% vs 17.1%), the incidence rate among cases exposed from household smoking was greater than that among no exposed cases (19.6% vs 14.3%), and the incidence rate among alcohol-drinkers was greater than that among non-drinkers (21.1% vs 15.1%). However, there were no statistically significant differences. Larger body mass index before pregnancy tended to be associated with the increased incidence rate of pre-eclampsia (p=0.160). Pregnant women with smoking had a statistically higher frequency of household smoking exposure and having drinking alcohol. Household smoking exposure and drinking alcohol status adjusted hazard rate ratio was 1.1 for smokers (95% confidence interval 0.6-1.7) as compared with that for non-smokers. Our results did not support the proposition that cigarette smoking protected women against pre-eclampsia.

Pre-eclampsia or eclampsia is a major contributor to pregnancy-related deaths at 20 weeks or more gestation.^1^ Considerable controversy exists over the etiology of pre-eclampsia. The following factors have been reported to play an important role in the development of this disease: increasing maternal age, working during pregnancy, multigravidity, nulliparity and obesity.^2^^-^^6^

Some attributes have been suggested which might protect women from developing pre-eclampsia. Maternal smoking has been reported to protect women against pre-eclampsia,^4^^,^^6^^-^^8^ although it is associated with medical complications such as spontaneous abortion, premature birth and delivering a small-for-date infant.^9^ However, the biologic mechanism by which cigarette smoking during pregnancy reduces the risk of pre-eclampsia is not certain. Although the etiology of pre-eclampsia is still unclear, several observations suggest that the maternal syndrome of pre-eclampsia has been ascribed to generalized maternal endothelial cell dysfunction and the endothelial dysfunction is a part of a more generalized intravascular inflammatory reaction involving intravascular leukocytes as well as the clotting and complement systems.^10^ Madretsma et al.^11^ reported that, in the vitro study, nicotine as well as prednisolone caused a significant inhibition of interleukin-2 and tumor necrosis factor-alpha production by human mononuclear cells. Cigarette smoking during pregnancy therefore might prevent the development of pre-eclampsia through nicotine-induced inhibition of interleukin-2 and tumor necrosis factor production.

In previous studies of cigarette smoking, there seemed to be two problems. First, a bias might be produced by misclassification of smoking habits: pregnant women were classified as non-smokers or smokers only by smoking status during early pregnancy, and if women quit smoking during the earlier stages of pregnancy, they were considered as non-smokers. Second, other lifestyles might be confounding factors: for example, cigarette smoking and alcohol drinking are closely associated with each other.

In this prospective cohort study, we tried to clarify the association between cigarette smoking and pre-eclampsia considering smoking status before pregnancy not to produce a bias by misclassification of smoking habits. Careful attention was paid to other lifestyles including alcohol drinking habits as confounding factors.

## METHODS

We conducted a prospective cohort study of pregnant women whose first antenatal visits were between September 1997 and April 1998 at Osaka Prefectural General Hospital, Japan. They were informed and consented to participation in this study. A self-administered questionnaire survey for lifestyles was carried out three times during pregnancy, before 14 weeks gestation, between 14 and 27 weeks gestation, and at/over 28 weeks gestation. Participants were asked about whether they were employed or not, smoked cigarettes, exposed to household smoking, and drank alcohol in each questionnaire. The questions concerning cigarette smoking and alcohol drinking status before pregnancy were also included in questionnaires carried out before 14 weeks gestation. Smokers/drinkers were defined considering smoking/alcohol drinking status before pregnancy: smokers were defined as persons who had smoked for the previous year, and drinkers were defined as persons who had drunk alcohol more than once per week for the previous year. Pregnancy outcome information was taken from medical record data. Cases who developed pre-eclampsia (WHO: International Statistical Classification of Diseases and Related Health Problems, 10th Revision; code O13-O14) conformed to the criteria for blood pressure and urinary protein shown in Table 1.

**Table 1.** Criteria for mild or severe pre-eclampsia.

Mild pre-eclampsia
A. Blood pressure (after 20 weeks gestation)
1. Systolic/diastolic blood pressure rises at least 30/15 mmHg from baseline value; or
2. Systolic/diastolic blood pressure rises at 140/90 mmHg or higher
B. Urinary protein (after 20 weeks gestation)
Two urinary protein dipstick measurements of 1+

Severe pre-eclampsia
A. Blood pressure (after 20 weeks gestation)
Systolic/diastolic blood pressure rises at 160 / 110 mmHg or higher
B. Urinary protein (after 20 weeks gestation)
Two urinary protein dipstick measurements of 3 to 4+

In International Statistical Classification of Diseases and Related Health Problems, 10th Revision, mild pre-eclampsia is included in gestational hypertension without significant proteinuria (O13), while severe pre-eclampsia is included in gestational hypertension with significant proteinuria (O14).

Of 527 subjects whose first antenatal visits were between September 1997 and April 1998, 34 subjects were excluded from the analysis because 2 subjects had spontaneous abortion and other 32 subjects were not followed. Total study subjects comprised 493 pregnant women who answered questionnaires carried out before 14 weeks gestation and were followed up until delivery. Of these, 360 pregnant women completed all questionnaires carried out in our study.

Categorical variables (i.e., characteristics and lifestyles) were assessed using Fisher’s exact tests and Mantel-extension chi-squares when appropriate. Differences in the incidence of pre-eclampia by smoking status were analyzed using the Cox’s proportional hazard model after adjustment for household smoking exposure and alcohol drinking. Body mass index was defined as weight (kg) divided by the square of the height (m). Differences were considered as statistically significant if p values were less than 0.05 by two-sided tests. Statistical package software, SPSS^®^ (version 10) was used for statistical analysis.

## RESULTS

Total study subjects were 493 pregnant women, which corresponded to 93.5% of the original cohort. Of the 493 subjects, 82 cases (16.6%) developed mild pre-eclampsia and 3 cases (0.6%) developed severe pre-eclampsia. Of the 82 patients who received diagnoses of mild pre-eclampsia, 74 conformed only to the blood pressure criteria.

**Figure 1.**
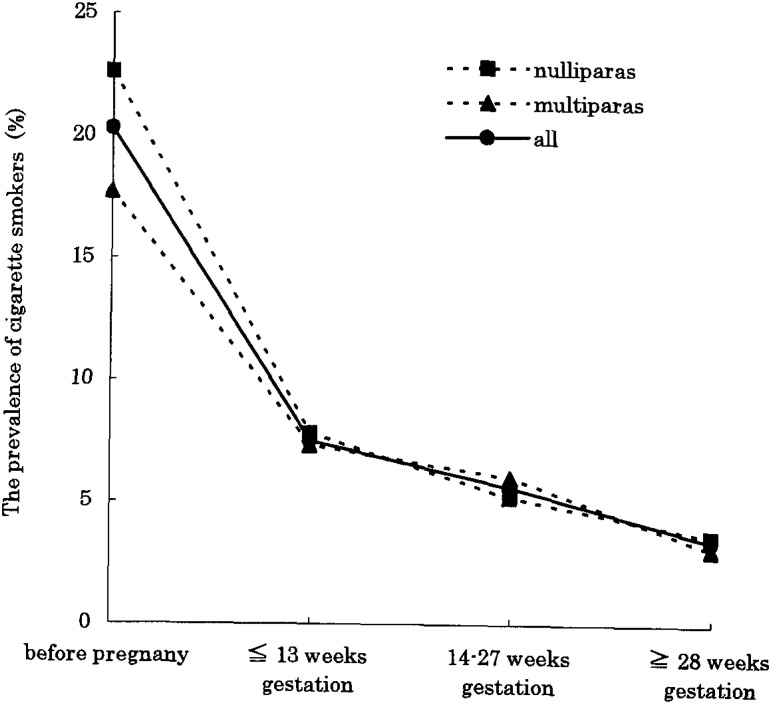
Trends in cigarette smoking during pregnancy. The horizontal axis shows the stages of investigation, and the vertical axis shows the prevalence of cigarette smokers.

Many pregnant women smoking before pregnancy had quit smoking during pregnancy. Among the 360 pregnant women who completed all questionnaires, the prevalence of cigarette smokers decreased from 20.3% to 3.4% during pregnancy. Three quarters (75.7%) of the women who had quit while pregnancy indicated that they had quit during first 14 weeks gestation (Figure 1).

Table 2 shows the association between variables (i.e., characteristics and lifestyles) and the incidence of pre-eclampsia. Almost the same incidence rate was observed among women aged less than 27 years as compared with women aged 31 years and over, among nulligravidas as compared with multigravidas, and among nulliparas as compared with multiparas. The incidence rate in housewives was greater than that in workers (62 of 317 [19.6%] vs 23 of 176 [13.1%], p=0.081).

**Table 2.** The association between variables and the incidence of pre-eclampsia. ^†^

	No. of study subjects	Incidence of pre-eclampsia	Incidence rates (%)	p value
Age (year)				
<28	171	28	16.4	0.802
28-30	161	29	18.0	
>30	161	28	17.4	
Work				
Housewives	317	62	19.6	0.081
Workers	176	23	13.1	
Gravidity				
1	225	40	17.8	0.811
2 or more	268	45	16.8	
Parity				
0	272	46	16.9	0.905
1 or more	221	39	17.6	
Cigarette smoking				
Non-smokers	380	65	17.1	0.563
Smokers	103	20	19.4	
Household smoking exposure				
No exposed	223	32	14.3	0.150
Exposed	270	53	19.6	
Alcohol drinking				
Non-drinkers	317	48	15.1	0.104
Drinkers	171	36	21.1	
Body mass index				
Lean (<18.5)	90	13	14.4	
Normal (18.5-25)	369	64	17.3	0.160
Overweight and Obese (>25)	29	8	27.6	

† In age and body mass index, Mantel-extension chi-square is used for examining differences in the incidence of pre-eclampsia. In others, Fisher’s exact test is used for examining differences in the incidence of pre-eclampsia.

The incidence rate among smokers was slightly greater than that among non-smokers (20 of 103 [19.4%] vs 65 of 380 [17.1%]), and the incidence rate among cases exposed from household smoking was greater than that among no exposed cases (53 of 270 [19.6%] vs 32 of 223 [14.3%]). The incidence rate among drinkers was greater than that among non-drinkers (36 of 171 [21.1%] vs 48 of 317 [15.1%]). Large body mass index before pregnancy tended to be associated with the increased incidence rate of pre-eclampsia (p=0.160). There were, however, no statistically significant differences.

The association between variables (i.e., characteristics and lifestyles) and prevalence of smokers are presented in Table 3. Pregnant women with smoking had a statistically higher frequency of household smoking exposure and having drinking alcohol. Household smoking exposure and drinking alcohol status adjusted hazard rate ratio was 1.1 for smokers (95% confidence interval 0.6-1.7) as compared with that for non-smokers, although the difference was not statistically significant.

**Table 3.** The association between variables and prevalence of smokers. ^†^

	No. of study subjects	No. of smokers	Prevalence of smokers (%)	p value
Age (year)				
<28	168	47	28.0	0.057
28-30	156	25	16.0	
>30	159	31	19.5	
Work				
Housewives	314	68	21.7	0.907
Workers	169	35	20.7	
Gravidity				
1	222	53	23.9	0.221
2 or more	261	50	19.2	
Parity				
0	266	65	24.4	0.074
1 or more	217	38	17.5	
Household smoking exposure				
No exposed	214	19	8.9	<0.01
Exposed	269	84	31.2	
Alcohol drinking				
Non-drinkers	311	50	16.1	<0.01
Drinkers	171	53	31.0	
Body mass index				
Lean (<18.5)	86	19	22.1	
Normal (18.5-25)	363	77	21.2	0.844
Overweight and Obese (>25)	29	6	20.7	

† In age and body mass index, Mantel-extension chi-square is used for examining differences in prevalence of smokers. In others, Fisher’s exact test is used for examining differences in prevalence of smokers.

## DISCUSSION

We investigated whether cigarette smoking was a protective factor against the development of pre-eclampsia with caution, because a bias might have been introduced by misclassification of smoking habits in previous studies. Furthermore, unlike past studies, we examined lifestyles as confounding factors.

Our results did not substantiate the concern that pre-eclampsia was caused by cigarette smoking, and indicated that cigarette smoking was not associated with the decreased incidence rate of pre-eclampsia: the incidence rate among smokers was somewhat greater than that among non-smokers. Conde-Agudelo et al.^8^ reported that maternal smoking was associated with a 32% reduction in the risk of pre-eclampsia, through the use of meta-analytic techniques, but the epidemiologic research on the reproductive consequences of cigarette smoking had been limited by the problems of reporting and misclassification biases in smoking status; cigarette consumption during pregnancy was not accurately reported because women tended to change their behaviors during early pregnancy (e.g., prevalence of cigarette smokers among our 493 pregnant women decreased from 21.3% to 8.6% during early pregnancy), many studies simply described women as smokers or non-smokers from results of questionnaires carried out during early pregnancy, and the quality of smoking data obtained was variable.

Considering household smoking exposure and alcohol drinking as confounding factors, our results also showed that cigarette smoking did not result in the significantly decreased incidence rate of pre-eclampsia. We need in the future to investigate using our definition of smokers and a comprehensive lifestyle analysis to reveal whether cigarette smoking with or without other factors causes pre-eclampsia, which will require a large number of study subjects in a cohort.

Recently an increased incidence of pre-eclampsia in Taiwanese women who worked during pregnancy was reported by Lee et al.^5^ We observed that women who worked during pregnancy were at lower risk of pre-eclampsia than women without that history (i.e. housewives), while gravidity and parity adjusted hazard rate ratio was 1.6 for workers (95% confidence interval 0.9-2.6, p=0.06) as compared with that for housewives. It is worth noting that gravidity and parity were confounding factors in the association between working status and the incidence of pre-eclampsia among Japanese women, but not office hours.

Our findings indicated that pregnant women changed their smoking behaviors easily during pregnancy: the prevalence of cigarette smokers before pregnancy in our study was almost the same as that among all Japanese women aged 20-39 years^12^ (the prevalence of cigarette smokers was 20.3% and 21.5% respectively), and it decreased from 20.3% to 3.4% during pregnancy. This figure of 3.4% is similar to the goal of the prevalence of cigarette smokers which was recognized by Osaka prefectural government in Healthy Osaka 21,^13^ which had been proposed to implement the goals of Healthy Japan 21 established by the Japanese Ministry of Health, Labor and Welfare. Fingerhut et al reported that 70% of women who had quit smoking during pregnancy resumed smoking within one year of delivery in the United States,^14^ therefore we should be highly sensitive to the danger of relapse. Personalized smoking cessation programs should be tailored to enable women to quit permanently in consideration of determinants of smoking and cessation during and after pregnancy (for example, maternal age, amount and duration of smoking, partner’s smoking habits, age of start smoking, parity and passive smoking).^15^ Permanent quitting by mothers will promote their children’s health because children of smoking parents are more likely to have adverse health outcomes such as respiratory illness than children of non-smoking parents.^16^^-^^18^ It will also prevent their children from starting cigarette smoking because poor health behavior in parents has substantial influence on their children and adolescents.^19^^,^^20^

Our findings do not support the contention that cigarette smoking reduces the risk of pre-eclampsia, and we argue for needs to provide programs to prevent pregnant women relapsing, in order to protect their children from the harmful effects of cigarette smoking.
